# Islet Autoantibody Measurements from Dried Blood Spots on Filter Paper Strongly Correlate to Serum Levels

**DOI:** 10.1371/journal.pone.0166213

**Published:** 2016-11-15

**Authors:** Kimber M. Simmons, Aimon K. Alkanani, Kristen A. McDaniel, Christopher Goyne, Dongmei Miao, Zhiyuan Zhao, Liping Yu, Aaron W. Michels

**Affiliations:** 1 Barbara Davis Center for Childhood Diabetes, University of Colorado, Aurora, CO, 80045, United States of America; 2 Indiana University School of Medicine, Indianapolis, IN, 46202, United States of America; University of Liverpool Institute of Infection and Global Health, UNITED KINGDOM

## Abstract

Type 1 diabetes (T1D) is increasing in incidence and predictable with measurement of serum islet autoantibodies (iAb) years prior to clinical disease onset. Identifying iAb positive individuals reduces diabetic ketoacidosis and identifies individuals for T1D prevention trials. However, large scale screening for iAb remains challenging as assays have varying sensitivities and specificities, insulin autoantibodies remain difficult to measure and venipuncture is generally required to obtain serum. We developed an approach to reliably measure all four major iAb, including insulin autoantibodies, from dried blood spots (DBS) on filter-paper. By spiking iAb positive serum into iAb negative whole blood in a dose titration, we optimized the conditions for autoantibody elution from filter paper as measured by fluid phase radioimmunoassays. After assessing stability of measuring iAb from DBS over time, we then screened iAb from DBS and the corresponding serum in new-onset T1D (n = 52), and controls (n = 72) which included first-degree relatives of T1D patients. iAb measured from eluted DBS in new-onset T1D strongly correlated with serum measurements (R^2^ = 0.96 for mIAA, GADA = 0.94, IA-2A = 0.85, ZnT8A = 0.82, p<0.01 for each autoantibody). There were no false positives in control subjects, and 5/6 with previously unknown iAb positivity in sera were detected using DBS. With further validation, measuring iAb from DBS can be a reliable method to screen for T1D risk.

## Introduction

The incidence of Type 1 diabetes (T1D), the immune mediated form of diabetes, has increased dramatically worldwide over the past two decades.[1] T1D can be identified prior to clinical onset of symptoms by measuring serum islet autoantibodies (iAb) to insulin (mIAA), glutamic decarboxylase (GADA), islet antigen (IA-2A), and zinc transporter (ZnT8A).[2] The presence of two or more iAb confers significant risk for disease development as approximately 70% of these children develop T1D within 10 years and have close to a 100% lifetime risk.[3] In Colorado, nearly half of children who develop T1D present in diabetic ketoacidosis, which is a life-threatening condition that occurs with prolonged insulin deficiency.[4] DKA confers significant risk for cerebral edema, functional changes in the brain, long-term neurocognitive deficits and even death.[5–7] The American Diabetes Association now recommends that relatives of patients with T1D be screened for islet autoimmunity through an available clinical research trial.[8] Monitoring for the development of islet autoimmunity and T1D in first-degree relatives (FDR) and individuals with high risk HLA-DQ-DR genotypes has been done in a number of prospective longitudinal studies with serial iAb measurements.[9–12] These individuals have a lower incidence of life-threatening diabetic ketoacidosis and also have the opportunity to participate in T1D prevention trials.[13–15] However, 85% of individuals who develop T1D do not have a family history and are not identified until they have clinical symptoms. Many prevention trials have focused on using preparations of insulin (subcutaneous, nasal and oral) in people at risk to delay T1D onset.[16, 17] Notably, a post-hoc analysis from the Diabetes Prevention Trial-Type 1 oral insulin trial found that treatment responders, defined by high titer insulin autoantibodies prior to treatment, had delayed progression to T1D.[18] A subsequent trial is underway to replicate these findings. iAb are routinely measured from serum obtained by venipuncture, which places limitations on large scale screening for early T1D identification.

Most successful and widely integrated screening and disease surveillance programs obtain samples from dried blood spots (DBS) on filter paper. The use of DBS to screen newborn infants for congenital metabolic disease has been in place for over fifty years.[19] DBS are now routinely used to screen for diseases such as hepatitis and HIV in developing countries.[20] To date, success in measuring iAb from DBS on filter paper has been limited, and no study has successfully measured all four major iAb from DBS on filter paper.[21, 22] However, a recent study conducted through the National Institute of Health sponsored TrialNet Pathway to Prevention Study collected both DBS and serum for iAb determination using fluid-phase radioimmunoassays (RIA) and found good concordance between DBS and serum measurements for GADA, IA-2A, and ZnT8A; however, mIAA were not measured [23]. Insulin autoantibodies have historically been difficult to measure in serum obtained from venipuncture and especially by alternative collection methods. Nevertheless, the need exists to measure insulin autoantibodies as they are often the first autoantibody to appear in young children, higher levels correlate with a shorter time to clinical disease onset and many prevention trials have focused on insulin therapies [24–27].

In the current study, we optimized the conditions to elute autoantibodies from DBS on filter paper, assessed stability over time, and then measured iAb in new-onset T1D patients (n = 52) and screened for iAb in controls (n = 72), which included both healthy individuals and FDRs. Eluted DBS iAb measurements were compared to serum measurements using fluid-phase RIAs. We report the reliable measurement of all four iAb, including insulin autoantibodies, from DBS on filter paper.

## Materials and Methods

### Subjects

The clinical investigation in this study was conducted in accordance with the Declaration of Helsinki principles. Study approval was provided by the Colorado Multiple Institutional Review Board, and written informed consent was received from participants prior to inclusion in the study. Subjects were recruited from the Barbara Davis Center for Diabetes clinics. New-onset T1D subjects were recruited at diagnosis or during standard follow-up visits. Controls were healthy volunteers and FDRs with an unknown iAb status.

#### Serum iAb measurements

Biochemical autoantibodies (mIAA, GADA, IA-2A, ZnT8A) were measured with fluid-phase RIAs at the Barbara Davis Center as previously described.[28–30] The mIAA assay was performed with a competition RIA. Levels of autoantibodies are expressed as a DK unit (NIDDK unit) from a standard curve for GADA and IA-2A or an index for mIAA and ZnT8A [index = (CPM sample-CPM negative control)/(CPM positive control-CPM negative control)] where CPM is counts per minute, with each antibody having its own positive standard for calculations. The intra-assay coefficient of variance for mIAA is 5.2%, GADA 4.9%, IA-2A 4.3% and ZnT8A 3.2%. The inter-assay coefficient of variance for mIAA is 16%, GADA 8.3%, IA-2A 9.9% and ZnT8A 10.4%. Positive cut-off values for serum RIA were index 0.010 for mIAA, 20 DK units for GADA, 5 DK units for IA-2A, and index 0.020 for ZnT8A, which are accepted and well validated cut-offs used to define iAb positivity by RIA both clinically and in large clinical research trials such as those conducted through Type 1 Diabetes TrialNet.

### iAb measurements from DBS on filter paper

For experiments optimizing iAb measurement from DBS on filter paper, high titer iAb positive serum was spiked into iAb negative whole blood, placed onto Whatman #903 filter paper and allowed to completely dry. This created an autoantibody dose titration from DBS on filter paper with the corresponding serum isolated for each condition. To elute iAb, two 6mm spots were punched from each DBS, and a single punch was placed in each well of a plastic 96-well flat bottom non-protein binding plate (Corning). Elution buffer (65 μl of 20mM Tris-HCl (pH 7.4), 150mM NaCl, 0.1% bovine serum albumin, 0.15% Tween-20 and 0.1% NaN_3_) was added to each well, and the plate was agitated for 30 minutes at room temperature and then incubated overnight at 4°C. Eluent from the DBS was stored at -20°C until analysis for iAb using fluid-phase RIA. Each 6mm punch from a DBS was used to measure a single iAb with 30 μl of eluent required for GADA, IA-2A, and ZnT8A RIA and 40 μl of eluent for mIAA. Fluid-phase RIAs, as described above, were used to measure iAb from both serum and DBS eluent.

Stability testing was conducted in a similar manner in which iAb positive serum was spiked into iAb negative whole blood and placed onto filter paper. Three DBS samples of varying iAb concentrations were prepared for each iAb and eluted weekly over a 28 day period.

For new-onset T1D and control subjects, fresh whole blood was collected from patients via venipuncture and 75 μl of blood was applied to the center of a 1.3 cm circle on filter paper; serum was obtained from the remaining whole blood. The blood spots were dried on an open surface at room temperature and stored for up to 4 weeks. Eluent from a single 6mm DBS punch was used to measure each iAb with a RIA. The intra-assay coefficient of variance from eluted DBS samples for mIAA is 9.9%, GADA 7.9%, IA-2A 9.7% and ZnT8A 9.8%. The inter-assay coefficient of variance for mIAA is 14.5%, GADA 9.7%, IA-2A 11.3% and ZnT8A 6.8%, which are similar to those for serum RIA.

### Statistical Analysis

Linear regression analysis was done to compare serum iAb levels to those measured from DBS on filter paper using GraphPad Prism 6.0 software. A two-tailed p value of <0.05 is considered significant. To determine sensitivity and specificity, positive cut-off values for serum were applied to DBS measurements.

## Results

### Measuring islet autoantibodies from dried blood spots on filter paper

**Fig 1:**
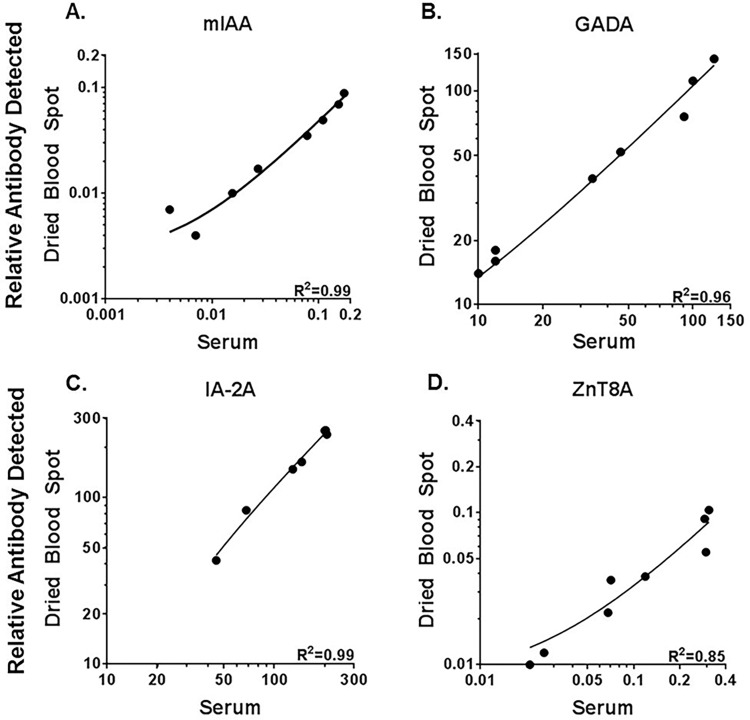
Islet autoantibody titrations comparing serum to dried blood spot eluent measurements. High titer autoantibody positive serum for each iAb was spiked into autoantibody negative whole blood in a dose titration. Blood spots were placed on filter paper and serum isolated for each titration. iAb were measured by serum radioimmunoassays for both DBS eluent and serum. Correlation of serum to DBS for A. insulin autoantibody (mIAA), B. glutamic decarboxylase autoantibody (GADA) C. protein tyrosine phosphatase insulinoma-associated-2 autoantibody (IA-2A), D. zinc transporter 8 autoantibody (ZnT8A). R^2^ is 0.99 mIAA, 0.96 GADA, 0.99 IA-2, 0.85 ZnT8; p<0.001 for all antibodies. The data is representative of three independent experiments.

To optimize and ensure successful iAb measurement from DBS on filter paper, high titer iAb positive serum was spiked into iAb negative whole blood to create a dose titration for each autoantibody. The iAb spiked blood was then placed on filter paper, and the corresponding serum isolated. Fluid-phase RIAs were used to measure iAb from both serum and DBS eluent. These experiments allowed for the evaluation of multiple variables including: eluent used for extraction, volume of eluent, plastic plate (standard, low protein binding, non-protein binding), number of DBS per well for protein extraction, incubation time in eluent, and agitation time to extract protein from filter paper. Using a non-protein binding plate, decreasing the volume of eluent per well, and agitating the sample prior to incubation optimized the measurement of iAb from DBS. Using optimized conditions, DBS iAb measurements correlated well with the corresponding serum measurements for each iAb as depicted in Fig 1 (R^2^ = 0.99 for mIAA, GADA = 0.96, IA-2A = 0.99 and ZnT8A = 0.85 with p<0.01 for each antibody).

**Fig 2:**
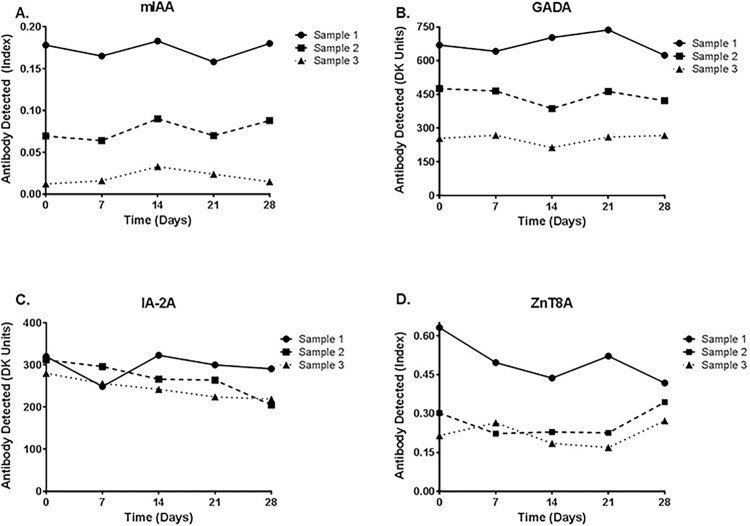
Stability of islet autoantibody measurements from dried blood spots over time. Islet autoantibody positive serum for each antibody was spiked into autoantibody negative whole blood, resulting in three separate samples of varying levels. Blood spots from each autoantibody sample were placed on filter paper and stored at room temperature. Samples were eluted from DBS at weekly intervals over a month and measured with fluid-phase RIAs. Levels for A. insulin autoantibody (mIAA), B. glutamic decarboxylase autoantibody (GADA), C. protein tyrosine phosphatase insulinoma-associated-2 autoantibody (IA-2A), D. zinc transporter 8 autoantibody (ZnT8A) were detectable over a 28-day time period for each sample.

We next evaluated the stability of measuring iAb from DBS on filter paper over time. Similar to the spiking experiments used to optimize the elution and measurement of iAb from DBS, we made three samples for each individual iAb and placed the samples on filter paper. The DBS were stored at room temperature and each sample was eluted at weekly intervals over a month. As shown in Fig 2, iAb levels remain stable as DBS on filter paper over a 28 day time period.

### Dried blood spot measurements in new-onset T1D and controls

**Fig 3:**
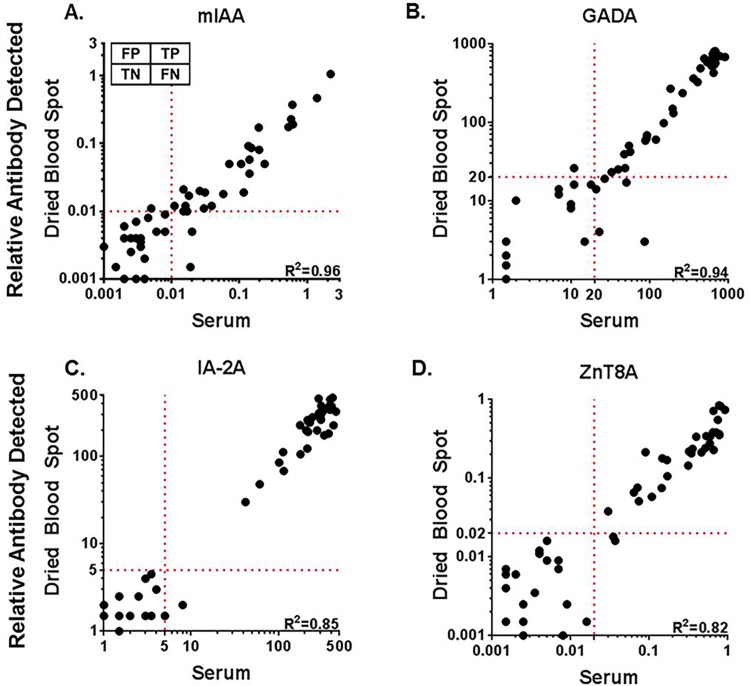
Comparison of islet autoantibodies in new-onset T1D patients measured from serum and dried blood spot eluent. Islet autoantibodies were measured in new-onset T1D patients (n = 52) from serum and dried blood spots obtained from the same blood sample by radioimmunoassay. Compared to serum A. insulin autoantibodies (mIAA) were detected in 27/29, B. glutamic acid decarboxylase antibodies (GADA) were detected in 35/39, C. protein tyrosine phosphatase insulinoma-associated-2 antibodies (IA-2A) were detected in 34/35, and D. zinc transporter 8 antibodies (ZnT8A) were detected in 32/33 DBS samples. FP = false positive, TP = true positive, TN = true negative, FN = false negative. Dotted lines indicate the level for positivity for each autoantibody; cut off values for serum was applied to DBS eluent levels. The linear correlation coefficient (R^2^) is depicted in the lower right hand corner for each iAb; p<0.001 for all antibodies.

After establishing a method to measure iAb from DBS on filter paper, we measured iAb from both DBS on filter paper and serum from new-onset T1D patients (n = 52). Table 1 describes the demographics of this cohort, which includes predominantly adolescents (median age 14). The median time from T1D onset was 13 days, and the majority of subjects (71%) had samples collected within 3 weeks of initiating insulin therapy. 90% of new onset T1D patients had ≥1 iAb present and 81% had ≥2 iAb present. As shown in Fig 3, serum measurements for each autoantibody significantly correlate with DBS measurements with R^2^ = 0.96 for mIAA, GADA 0.94, IA-2A 0.85, and ZnT8A 0.82. The standard positive cut-off values for serum were applied to DBS measurements. Insulin autoantibodies were present in 29 of 52 new-onset patients, and DBS measurements detected 27/29 (93%). DBS detected 35/39 (90%) GADA, 34/35 (97%) IA-2A and 32/33 (97%) ZnT8A in the new-onset patients. It was predominantly low-level iAb in the serum that was not detected from DBS. For each iAb, both sensitivity (93% for mIAA, GADA 90%, IA-2A 97%, ZnT8A 97%) and specificity (100% for mIAA, GADA 92%, IA-2A 100%, ZnT8A 100%) measured from DBS compared to serum were acceptably high. This resulted in very high positive predictive values (93% for mIAA, GADA 97%, IA-2A 100%, ZnT8A 100%) and negative predictive values (91% for mIAA, GADA 75%, IA-2A 94%, ZnT8A 95%).

**Table 1:** Clinical characteristics and islet autoantibodies in study.

	New onset T1D (n = 52)	Controls^a^ (n = 72)
Age (years)		
median	14	33
mean	16	33
range	4–41	9–77
T1D Duration (days)		
median	13	—
mean	43	—
range	0–540	—
Sex		
% males	62%	38%
Race		
White	86%	78%
Black	8%	1%
Hispanic	5%	11%
Other	1%	10%
Antibody Positivity by serum RIA^b^		
Insulin	56% (29/52)	3% (2/72)
GADA	75% (39/52)	6% (4/72)
IA-2A	67% (35/52)	3% (2/72)
ZnT8A	63% (33/52)	1% (1/72)
≥2 islet autoantibodies	81% (42/52)	3% (2/72)
≥1 islet autoantibodies	90% (47/52)	7% (5/72)

^a^controls include first degree relatives (n = 34)

^b^RIA = radioimmunoassay

**Fig 4:**
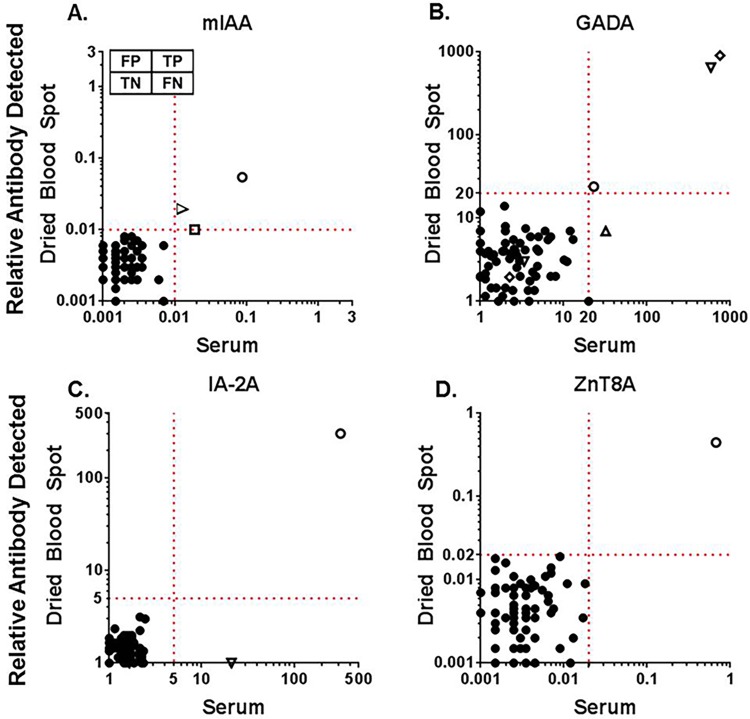
Comparison of islet autoantibodies in control patients measured from serum and dried blood spot eluent. Islet autoantibodies were measured in control patients (n = 72), which include both first-degree relatives and healthy controls, from serum and dried blood spots obtained from the same blood sample by radioimmunoassay. There were no false positive DBS measurements. A. insulin autoantibody (mIAA), B. glutamic decarboxylase autoantibody (GADA), C. protein tyrosine phosphatase insulinoma-associated-2 autoantibody (IA-2A), D. zinc transporter 8 autoantibody (ZnT8A). FP = false positive, TP = true positive, TN = true negative, FN = false negative. Open symbols denote controls positive for at least one iAb; matching open symbols are from the same individual.

We then screened iAb from both DBS on filter paper and serum in healthy control subjects (n = 72) in which 34 were FDRs. Since FDRs have a higher risk for T1D than normal controls, FDRs were included in an attempt to identify individuals otherwise not known to have iAb positivity. Normal controls helped determine the likelihood of false positive iAb measurements. Table 1 includes the demographic data in which controls had a median age of 33. As depicted in Fig 4, there were no false positive DBS measurements for each iAb. Positive iAb were detected in 6 controls by serum RIA measurements. Two subjects were positive for mIAA alone by both serum RIA (index 0.019, 0.021) and DBS (index 0.010, 0.019). Another subject was GADA positive, again detected by DBS measurement. One subject was positive for all four iAb by both methods. A subject with a low level positive GADA by serum (32 DK units) was not identified by DBS. A final control subject was positive for both GADA and IA-2A by serum RIA but only positive for GADA by DBS measurement. Again, the IA-2A level was at the lower level of positivity.

## Discussion

Our results demonstrate that iAb measurements from DBS on filter paper strongly correlate to serum levels. Importantly, we were able to measure insulin autoantibodies from DBS with a significant correlation to RIA serum measurements, which has historically been challenging. Our ability to successfully measure mIAA from filter paper was influenced by several factors. First, we optimized the assay by using a non-protein binding plate to limit the binding of antibody to the plate. We also determined the minimum sample volume necessary to measure all four iAb, thereby concentrating iAb in elution buffer. Finally, agitating the filter-paper samples in elution buffer improved protein elution. In addition to variables we manipulated, part of our success is due to the fact that mIAA assays and measurement have improved greatly over the last decade through the Diabetes Antibody Standardization Program [31].

Reliably measuring mIAA is important for several reasons. First, young children can be identified in the pre-symptomatic stage of T1D as mIAA are often the first iAb to develop and mIAA levels correlate with time to disease onset.[24–27] Second, there is an eligibility requirement to be mIAA positive to enroll in current and upcoming oral insulin prevention trials (NCT00419562, NCT02580877). With the ability to reliably measure mIAA from DBS on filter paper, identifying young children in the early stages of T1D becomes feasible and the approach is adaptable for general population screening. It is noteworthy that one group reported good concordance between insulin antibody titers using dried capillary blood spots and serum; however, only 15 T1D subjects in their cohort were positive for insulin antibodies.[22] Our results need to be validated in a larger cohort of patients using a capillary finger-stick to obtain DBS followed by a confirmatory serum test on patients with positive iAb before DBS methodology can be widely adopted as a screening test.

Although it is important that screening tests have high sensitivity, it is also necessary to limit the false positive rate to avoid patient and economic burden. In our control group, there were no false positive measurements. We did identify 5 of 6 control subjects by DBS who had at least one positive serum iAb. The iAb not detected by DBS was near the lower limit of detection in serum. Following surveillance models used in other diseases, a screening test requires a gold standard confirmatory test. Therefore, although DBS may provide a method for first-line screening, patients who are positive require a confirmatory test with serum for iAb measurement. Using DBS on filter paper offers distinct advantages for large scale early identification of T1D risk. DBS are easy to collect and store, stable over time, easily transported by mail to a core laboratory that is proficient in measuring iAb, cost effective and have a low infectivity risk. Limitations to using DBS to measure iAb include the need to have a completely dried spot on filter paper, elution of samples for analysis, small volumes are obtained following elution and hemolysis may potentially affect results.

Measuring iAb from DBS on filter paper has the potential to be a viable alternative to serum measurements in a variety of clinical and research environments including pediatric practices, rural areas and developing countries. Now that it is feasible to measure the four conventional iAb including insulin from a minimal amount of blood dried on filter paper, validation studies are warranted to expand this approach into a general population screening test for T1D risk.

## References

[1] Atkinson MA, Eisenbarth GS, Michels AW. Type 1 diabetes. The Lancet. 2014;383(9911):69–82. 10.1016/s0140-6736(13)60591-7 PMC4380133 23890997

[2] Insel RA, Dunne JL, Atkinson MA, Chiang JL, Dabelea D, Gottlieb PA, et al Staging Presymptomatic Type 1 Diabetes: A Scientific Statement of JDRF, the Endocrine Society, and the American Diabetes Association. Diabetes care. 2015;38(10):1964–74. 10.2337/dc15-1419 . 26404926 PMC5321245

[3] Ziegler AG, Rewers M, Simell O, Simell T, Lempainen J, Steck A, et al Seroconversion to multiple islet autoantibodies and risk of progression to diabetes in children. JAMA: the journal of the American Medical Association. 2013;309(23):2473–9. 10.1001/jama.2013.6285 . 23780460 PMC4878912

[4] Rewers A, Dong F, Slover RH, Klingensmith GJ, Rewers M. Incidence of diabetic ketoacidosis at diagnosis of type 1 diabetes in Colorado youth, 1998–2012. JAMA: the journal of the American Medical Association. 2015;313(15):1570–2. 10.1001/jama.2015.1414 . 25898057

[5] Cameron FJ, Scratch SE, Nadebaum C, Northam EA, Koves I, Jennings J, et al Neurological consequences of diabetic ketoacidosis at initial presentation of type 1 diabetes in a prospective cohort study of children. Diabetes care. 2014;37(6):1554–62. 10.2337/dc13-1904 24855156 PMC4179516

[6] Lin A, Northam EA, Werther GA, Cameron FJ. Risk Factors for Decline in IQ in Youth With Type 1 Diabetes Over the 12 Years From Diagnosis/Illness Onset. Diabetes care. 2015;38(2):236–42. 10.2337/dc14-1385 . 25488913

[7] Semenkovich K, Bischoff A, Doty T, Nelson S, Siller AF, Hershey T, et al Clinical presentation and memory function in youth with type 1 diabetes. Pediatric diabetes. 2015 10.1111/pedi.12314 26377697 PMC4803626

[8] American Diabetes A. Standards of Medical Care in Diabetes-2016 Abridged for Primary Care Providers. Clin Diabetes. 2016;34(1):3–21. 10.2337/diaclin.34.1.3 . 26807004 PMC4714725

[9] Ziegler AG, Hummel M, Schenker M, Bonifacio E. Autoantibody appearance and risk for development of childhood diabetes in offspring of parents with type 1 diabetes: the 2-year analysis of the German BABYDIAB Study. Diabetes. 1999;48(3):460–8. . 10078544 10.2337/diabetes.48.3.460

[10] Kupila A, Muona P, Simell T, Arvilommi P, Savolainen H, Hamalainen AM, et al Feasibility of genetic and immunological prediction of type I diabetes in a population-based birth cohort. Diabetologia. 2001;44(3):290–7. . 11317658 10.1007/s001250051616

[11] Barker JM, Barriga KJ, Yu L, Miao D, Erlich HA, Norris JM, et al Prediction of autoantibody positivity and progression to type 1 diabetes: Diabetes Autoimmunity Study in the Young (DAISY). The Journal of clinical endocrinology and metabolism. 2004;89(8):3896–902. 10.1210/jc.2003-031887 . 15292324

[12] Krischer JP, Lynch KF, Schatz DA, Ilonen J, Lernmark A, Hagopian WA, et al The 6 year incidence of diabetes-associated autoantibodies in genetically at-risk children: the TEDDY study. Diabetologia. 2015;58(5):980–7. 10.1007/s00125-015-3514-y 25660258 PMC4393776

[13] Winkler C, Schober E, Ziegler AG, Holl RW. Markedly reduced rate of diabetic ketoacidosis at onset of type 1 diabetes in relatives screened for islet autoantibodies. Pediatric diabetes. 2012;13(4):308–13. 10.1111/j.1399-5448.2011.00829.x . 22060727

[14] Elding Larsson H, Vehik K, Bell R, Dabelea D, Dolan L, Pihoker C, et al Reduced prevalence of diabetic ketoacidosis at diagnosis of type 1 diabetes in young children participating in longitudinal follow-up. Diabetes care. 2011;34(11):2347–52. 10.2337/dc11-1026 21972409 PMC3198296

[15] Triolo TM, Chase HP, Barker JM, Group DPTS. Diabetic subjects diagnosed through the Diabetes Prevention Trial-Type 1 (DPT-1) are often asymptomatic with normal A1C at diabetes onset. Diabetes care. 2009;32(5):769–73. 10.2337/dc08-1872 19407074 PMC2671125

[16] Diabetes Prevention Trial—Type 1 Diabetes Study G. Effects of insulin in relatives of patients with type 1 diabetes mellitus. The New England journal of medicine. 2002;346(22):1685–91. 10.1056/NEJMoa012350 . 12037147

[17] Bonifacio E, Ziegler AG, Klingensmith G, Schober E, Bingley PJ, Rottenkolber M, et al Effects of high-dose oral insulin on immune responses in children at high risk for type 1 diabetes: the Pre-POINT randomized clinical trial. JAMA: the journal of the American Medical Association. 2015;313(15):1541–9. 10.1001/jama.2015.2928 . 25898052

[18] Skyler JS, Krischer JP, Wolfsdorf J, Cowie C, Palmer JP, Greenbaum C, et al Effects of oral insulin in relatives of patients with type 1 diabetes: The Diabetes Prevention Trial—Type 1. Diabetes care. 2005;28(5):1068–76. . 15855569 10.2337/diacare.28.5.1068

[19] Guthrie R, Susi A. A Simple Phenylalanine Method for Detecting Phenylketonuria in Large Populations of Newborn Infants. Pediatrics. 1963;32:338–43. . 14063511

[20] Demirev PA. Dried blood spots: analysis and applications. Analytical chemistry. 2013;85(2):779–89. 10.1021/ac303205m . 23171435

[21] Bazzigaluppi E, Bonfanti R, Bingley PJ, Bosi E, Bonifacio E. Capillary whole blood measurement of islet autoantibodies. Diabetes care. 1999;22(2):275–9. . 10333945 10.2337/diacare.22.2.275

[22] Siraj ES, Rogers DG, Gupta MK, Reddy SS. A simple screening method for individuals at risk of developing type 1 diabetes: measurement of islet cell autoantibodies (GADA, IA-2A, and IAA) on dried capillary blood spots collected on filter paper. Hormone and metabolic research = Hormon- und Stoffwechselforschung = Hormones et metabolisme. 2012;44(11):855–60. 10.1055/s-0032-1316349 . 22893260

[23] Bingley PJ, Rafkin LE, Matheson D, Steck AK, Yu L, Henderson C, et al Use of Dried Capillary Blood Sampling for Islet Autoantibody Screening in Relatives: A Feasibility Study. Diabetes technology & therapeutics. 2015;17(12):867–71. 10.1089/dia.2015.0133 26375197 PMC4677115

[24] Dean BM, Becker F, McNally JM, Tarn AC, Schwartz G, Gale EA, et al Insulin autoantibodies in the pre-diabetic period: correlation with islet cell antibodies and development of diabetes. Diabetologia. 1986;29(5):339–42. . 3522332 10.1007/BF00452073

[25] Simell S, Hoppu S, Simell T, Stahlberg MR, Viander M, Routi T, et al Age at development of type 1 diabetes- and celiac disease-associated antibodies and clinical disease in genetically susceptible children observed from birth. Diabetes care. 2010;33(4):774–9. 10.2337/dc09-1217 20056952 PMC2845026

[26] Steck AK, Johnson K, Barriga KJ, Miao D, Yu L, Hutton JC, et al Age of islet autoantibody appearance and mean levels of insulin, but not GAD or IA-2 autoantibodies, predict age of diagnosis of type 1 diabetes: diabetes autoimmunity study in the young. Diabetes care. 2011;34(6):1397–9. 10.2337/dc10-2088 21562325 PMC3114355

[27] Steck AK, Vehik K, Bonifacio E, Lernmark A, Ziegler AG, Hagopian WA, et al Predictors of Progression From the Appearance of Islet Autoantibodies to Early Childhood Diabetes: The Environmental Determinants of Diabetes in the Young (TEDDY). Diabetes care. 2015;38(5):808–13. 10.2337/dc14-2426 25665818 PMC4407751

[28] Yu L, Robles DT, Abiru N, Kaur P, Rewers M, Kelemen K, et al Early expression of antiinsulin autoantibodies of humans and the NOD mouse: evidence for early determination of subsequent diabetes. Proceedings of the National Academy of Sciences of the United States of America. 2000;97(4):1701–6. 10.1073/pnas.040556697 10677521 PMC26499

[29] Wenzlau JM, Juhl K, Yu L, Moua O, Sarkar SA, Gottlieb P, et al The cation efflux transporter ZnT8 (Slc30A8) is a major autoantigen in human type 1 diabetes. Proceedings of the National Academy of Sciences of the United States of America. 2007;104(43):17040–5. 10.1073/pnas.0705894104 17942684 PMC2040407

[30] Bonifacio E, Yu L, Williams AK, Eisenbarth GS, Bingley PJ, Marcovina SM, et al Harmonization of glutamic acid decarboxylase and islet antigen-2 autoantibody assays for national institute of diabetes and digestive and kidney diseases consortia. The Journal of clinical endocrinology and metabolism. 2010;95(7):3360–7. 10.1210/jc.2010-0293 20444913 PMC2928900

[31] Schlosser M, Mueller PW, Torn C, Bonifacio E, Bingley PJ, Participating L. Diabetes Antibody Standardization Program: evaluation of assays for insulin autoantibodies. Diabetologia. 2010;53(12):2611–20. 10.1007/s00125-010-1915-5 . 20871974

